# Mechanical Properties and Stress–Strain Relationship of PVA-Fiber-Reinforced Engineered Geopolymer Composite

**DOI:** 10.3390/polym16121685

**Published:** 2024-06-13

**Authors:** Jian Zhou, Zhenjun Li, Xi Liu, Xinzhuo Yang, Jiaojiao Lv

**Affiliations:** 1 Northwest Engineering Corporation Limited, Xi’an 710065, China; jianzhounec@163.com (J.Z.); yangxinzhuo123@126.com (X.Y.); lvjj@nwh.cn (J.L.); 2 School of Civil Engineering, Chang’an University, Xi’an 710064, China; lizhenjun0512@163.com

**Keywords:** engineering geopolymer composite, polyvinyl alcohol fiber, mechanical property, uniaxial tensile behaviors, stress–strain constitutive model, fractal dimension

## Abstract

In this study, seven Engineering Geopolymer Composite (EGC) groups with varying proportions were prepared. Rheological, compressive, flexural, and axial tensile tests of the EGC were conducted to study the effects of the water/binder ratio, the cement/sand ratio, and fiber type on its properties. Additionally, a uniaxial tension constitutive model was established. The results indicate that the EGC exhibits early strength characteristics, with the 7-day compressive strength reaching 80% to 92% of the 28-day compressive strength. The EGC demonstrates high compressive strength and tensile ductility, achieving up to 70 MPa and 4%, respectively. The mechanical properties of the EGC improved with an increase in the sand/binder ratio and decreased with an increase in the water/binder ratio. The stress–strain curve of the EGC resembles that of the ECC, displaying a strain-hardening state that can be divided into two stages: before cracking, the matrix primarily bears the stress; after cracking, the slope decreases, and the fiber predominantly bears the stress.

## 1. Introduction

Concrete structures are known for their resilience, durability, and sustainability limitations, primarily due to their propensity to crack under tension [1]. In response to this challenge, Engineered Cementitious Composites (ECCs) or Strain-Hardening Cementitious Composites (SHCCs) were introduced in the early 1990s by Li [2]. These materials, however, come with a significant drawback: a high cement content. ECCs require two to three times the amount of Ordinary Portland Cement compared to traditional concrete due to eliminating coarse aggregates from their mix design [3]. Producing every ton of cement releases approximately 0.7 to 1.0 tons of carbon dioxide [4,5], contributing to cement production accounting for 7% of global carbon emissions [6,7,8]. Consequently, the production costs and energy consumption of ECCs are substantially higher.

Geopolymer concrete has emerged as a promising alternative to Ordinary Portland Cement (OPC) concrete in various challenging environments in recent years. This innovative material boasts superior mechanical properties, enhanced durability, and excellent corrosion and heat resistance while significantly reducing carbon emissions [9]. Furthermore, geopolymer concrete presents an opportunity to convert diverse wastes into valuable construction materials, thereby diminishing greenhouse gas emissions associated with cement production [10]. Nonetheless, geopolymer concrete tends to be more brittle than its OPC counterpart, breaking more readily under tensile stress at equivalent strength levels [11]. A viable solution to mitigate this brittleness involves incorporating fiber-enhanced materials into geopolymer concrete, enhancing its structural integrity. This approach has led to the development of Engineering Geopolymer Composite (EGC) materials, characterized by their high ductility and capacity to develop multiple micro-cracks when subjected to loading [12,13]. Notably, EGC materials exhibit distinct strain-hardening behavior, significantly enhancing the deformability of geopolymers [14,15,16].

Research by Lee and Ohno [17,18] has highlighted the substantial improvements in the deformation capabilities of geopolymer concrete offered by EGC materials. Fernandez [19] observed that a fly-ash-based EGC demonstrated a remarkable elongation rate of 4.3%, albeit with a slight reduction in compressive and tensile strength. Wang [20] explored the impact of varying sand content on the engineering properties of an EGC, noting that specimens exhibited deflection hardening behavior and developed multiple surface cracks. Furthermore, Lao [21] designed and developed High-Strength Engineered Geopolymer Composites (HS-EGCs) dominated by fly ash (FA), and the HS-EGCs achieved high compressive strengths (over 100 MPa) and high tensile ductility (over 4%). Wang [22] developed a high-ductility, cement-free Engineered Geopolymer Composite (EGC) with a 0.2% fiber content (EGC-0.2%). The EGC-0.2% achieved over 4% tensile strain capacity, 200 µm crack width, 39 MPa compressive strength, and a density below 1200 kg/m³, resulting in a specific strength of 37 kPa/(kg/m³). Krevaikas and Ombres explored the impact of long fibers on the properties of cement-based composite materials, analyzing the mechanical properties of fiber-reinforced cementitious systems and textile-reinforced mortars and examining their role in enhancing structural performance [23,24].

To optimize the design and performance regulation of PVA-EGCs, in this study, the mechanical properties and stress–strain relationship of a PVA-EGC are investigated. The influences of the water/binder ratios, sand-to-binder ratios, and fiber type on the workability, mechanical strength, and uniaxial tensile behavior are studied. A constitutive model is proposed to evaluate this multifactor coupling mechanism and predict the stress–strain response of the GRC. The box-counting method was used to analyze the fractal characteristics of the EGC uniaxial tensile cracks, and the fractal dimension and ultimate stress and strain of the specifications were established. This study promotes the refinement and development of constitutive models for EGCs and lays the foundation for the practical application and engineering design of EGC materials and members.

## 2. Materials and Experiments

### 2.1. Materials

#### 2.1.1. Binders

**Figure 1:**
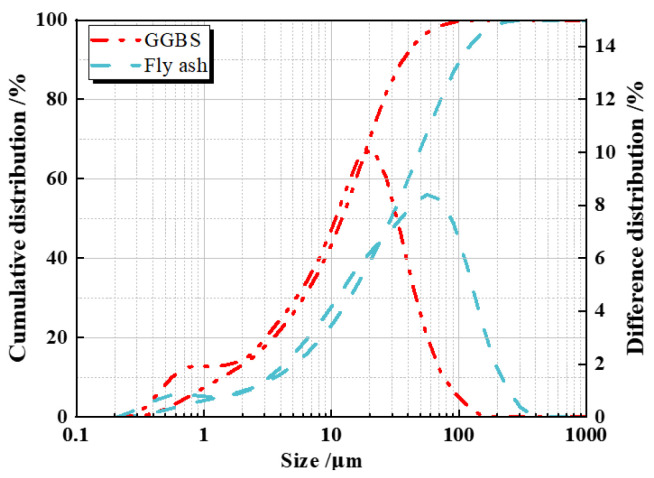
Particle size distribution of raw materials.

**Figure 2:**
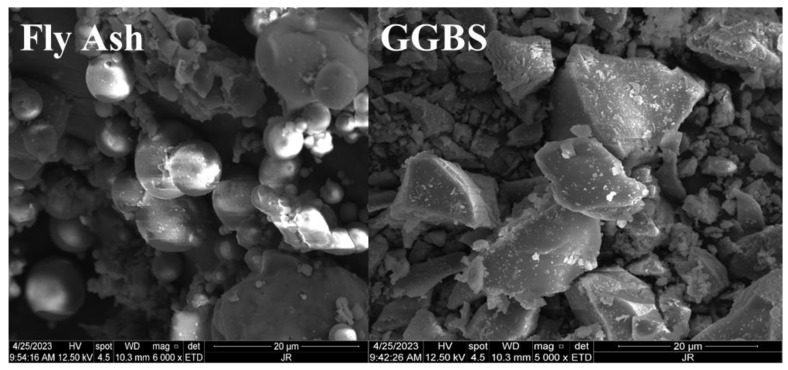
SEM images of raw materials.

Low-calcium fly ash (FA) and S95-grade Ground Granulated Blast Furnace Slag (GGBS) were chosen as the primary binders for this study. The chemical compositions of these raw materials are detailed in Table 1. The particle size distribution (PSD) of the FA and GGBS are presented in Figure 1. The average particle sizes for the FA and GGBS were 23.2 nm and 24.0 nm, respectively. Additionally, SEM images highlighting the microstructural characteristics of the fly ash and GGBS particles are depicted in Figure 2.

#### 2.1.2. Alkali Activator

The alkali activator solution was formulated using water glass, sodium hydroxide (NaOH), and water in specific proportions. Na_2_SiO_3_ and NaOH were sourced from Xi’an Huachang Water Glass Co., with the Na_2_SiO_3_ having a modulus of 2.8 and the NaOH being solid flakes with a purity of ≥98.0%. To prepare the solution, Na_2_SiO_3_ and NaOH were each dissolved in water to achieve a 40% mass fraction solution. These solutions were allowed to stand for 12 h to ensure complete dissolution. Subsequently, they were mixed according to predetermined proportions to produce an alkali activator solution with a final modulus of 1.6.

#### 2.1.3. Fiber and Superplasticizer

Table 2 summarizes the basic properties of the Polyvinyl Alcohol (PVA) fibers. This study compared PVA fibers from two sources: an REC15-type PVA fiber produced by Kuraray Co., Kakudacho, Kita-ku, Japan and a PVA fiber manufactured by Shanghai Yingjia Industrial Company, shanghai, China. The primary distinction between these fibers lies in their surface treatment methods. A comparative analysis of these fibers’ cost and performance was conducted to identify the most suitable option for our application.

#### 2.1.4. Quartz Sand

The properties of the quartz sand used in this study are presented in Table 3.

### 2.2. Mix Proportion and Specimen Preparation

This study designed seven mix proportions, varying the water/binder ratios (0.28, 0.30, and 0.32) and sand-to-binder ratios (0.3, 0.35, and 0.4) to evaluate their effects on the performance of Engineered Geopolymer Composites (EGCs). Additionally, we established a comparison group using domestic fibers to assess the impact of fiber type on EGC performance and to explore the potential for substituting Japanese Kuraray fibers. Another comparison group, Engineered Cementitious Composites (ECCs), was created to examine the performance differences between an EGC and ECC when mixed in identical ratios. The detailed mix proportions and comparison groups are presented in Table 4.

Initially, the required materials were weighed. The sodium hydroxide and sodium silicate solutions were mixed, while the water and water reducer were combined and stirred uniformly with a glass rod. PVA fibers, prone to clumping, were dispersed by hand.

Subsequently, fly ash, slag, and quartz sand were placed into the mixer and stirred at a low speed for 2 min to achieve a homogeneous mixture. The prepared alkali activator solution was then gradually added, stirring for 1 min. This was followed by adding the pre-mixed water and water-reducing agent, which were stirred vigorously for 2 min. The PVA fibers were evenly introduced in alignment with the mixer’s rotational direction to ensure their homogeneous dispersion throughout the mixture. High-speed stirring was continued for 4-6 min before the mixture was poured into molds.

The slurry was poured halfway into the mold, followed by vibration for 1 min to compact. After adding the remaining slurry, the vibration was continued for another minute. The specimen’s surface was then smoothed, and a plastic film was placed over it to prevent moisture loss. The mold was removed 24 h later.

For post-demolding treatment, specimens were placed in an oven at 60 °C for 24 h of high-temperature curing. Subsequently, they were transferred to a constant temperature and humidity room maintained at (20 ± 2) °C and (95 ± 2)% humidity until the testing age; at this point, the respective tests were conducted.

### 2.3. Test Methods

The flowability of the freshly mixed Engineered Geopolymer Composite (EGC) mortars was assessed using the table flow test, as specified in the Chinese standard GB/T 2419-2005 [25], “Method for Determining the Flowability of Cement Mortar”.

Each mix proportion uses nine cubic specimens measuring 70.7 × 70.7 × 70.7 mm^3^ to assess the compressive strength at various ages. These tests were performed on a 1000 kN electrohydraulic servo universal testing machine with loading rates set at 2.5 kN/s. Similarly, for the evaluation of flexural strength, each mix employs six prismatic specimens with dimensions of 40 mm × 40 mm × 160 mm, as stipulated by the same standard. The flexural strength tests were conducted using a TYP-3000 testing machine, which applies loads at 0.05 kN/s.

**Figure 3:**
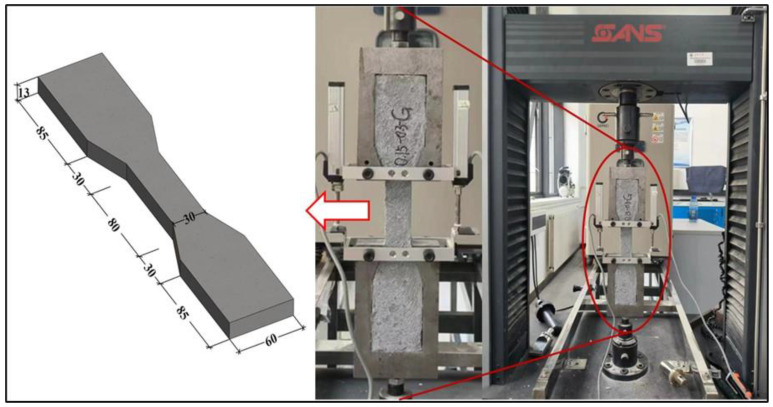
Uniaxial tensile testing.

For uniaxial tensile performance, the specifics of the specimen dimensions and testing apparatus are detailed in Figure 3. A minimum of three valid uniaxial tensile tests were conducted for each type of specimen. The measurement of the mid-specimen elongation was performed over a length of 80 mm. Detachable automatic resistance strain displacement sensors were affixed to both sides of the tensile specimen to improve accuracy and efficiency in data collection. The average value from the data collected is considered as the practical displacement. Each batch of tests ensures at least three valid datasets for tensile strength using a 50 kN SANS electronic universal testing machine at a 0.1 mm/min loading rate.

## 3. Experimental Results

### 3.1. Rheological Property

This section explores the rheological properties of the Engineered Geopolymer Composite (EGC) across different mixtures, with workability results detailed in Table 5. An increase in the sand-to-binder (S/B) ratio from 0.3 to 0.4 led to an 11.5% reduction in the EGC slurry flowability. This reduction can be attributed to the rise in large particle numbers and pore volume within the EGC matrix under constant water usage. Consequently, more water is required to fill these pores, diminishing the water available to coat particle surfaces and facilitate fluidity. This scarcity of surface water decreases the overall fluidity of the EGC matrix.

Furthermore, water consumption significantly influences the mix’s flow properties. As the water-to-binder (W/B) ratio increases, so does the fluidity of the EGC slurry. Specifically, when the W/B ratio rises from 0.28 to 0.32, fluidity increases from 14.8 cm to 18.3 cm. This enhancement is primarily due to the additional free water in the mix, which thickens the water film between particles, serving as a lubricant. Conversely, a low W/B ratio can reduce the EGC slurry’s flowability, hindering fiber dispersion.

### 3.2. Mechanical Strength

#### 3.2.1. Compressive Strength

**Figure 4:**
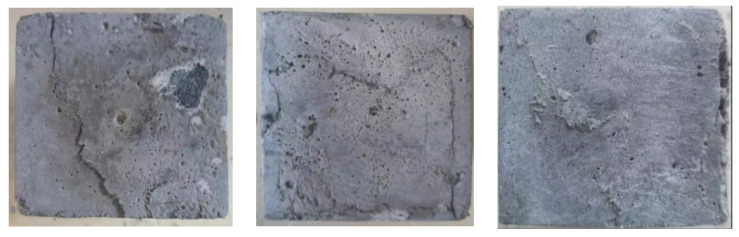
Failure mode.

Upon pressurization, the EGC specimens exhibited not only a retardation in the macroscopic development of initial cracks but also a limitation in the extension of longitudinal compression cracks. As the load increased, the Polyvinyl Alcohol (PVA) fibers were pulled out or broken. Concurrently, a hissing noise was observed, indicating that the test block’s plastic deformation was intensifying. The formation of new micro-cracks gradually released energy, developing multiple fine cracks within the test block. This phenomenon demonstrated the material’s considerable compressive toughness. After reaching the peak load, it decreased slowly, and the EGC specimens remained intact, without any visible bulging or spalling, showcasing their damaged morphology as depicted in Figure 4.

**Figure 5:**
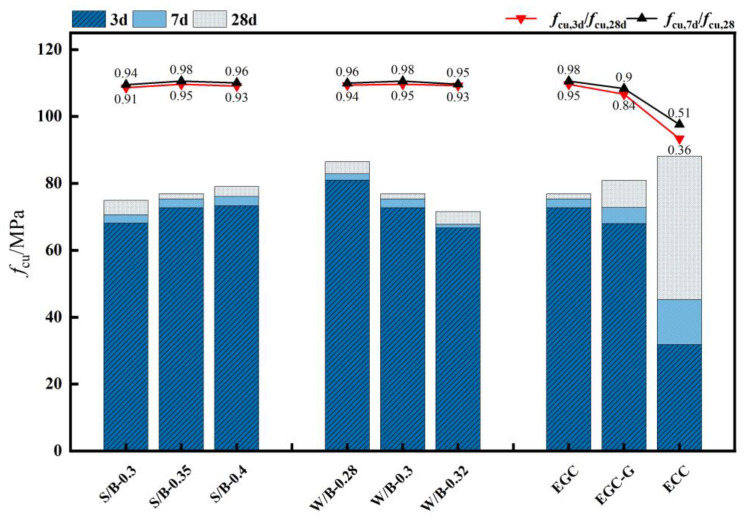
Compressive strength.

Figure 5 and Table 6 illustrate the relationship between the 3-day, 7-day, and 28-day compressive strengths of a high-ductility geopolymer concrete with different mix proportions. The 3-day and 7-day compressive strengths of the EGCs reached 81%96% and 90%98% of the 28-day strength, respectively, significantly higher than the cement-based ECC. This is mainly due to the rapid early strength development of geopolymer materials compared to ordinary cement-based materials and the 24 h curing at 60 °C before standard curing, which significantly increased the compressive strength of the high fly ash content specimens.

The EGC specimens maintained their integrity post-compression and were devoid of external bulging or spalling. This reveals that an increase in the sand-to-binder ratio results in a compressive strength increase from 74.92 MPa to 79.08 MPa, marking a 5.55% enhancement. This suggests that a higher sand-to-binder ratio positively influences the EGC’s compressive strength within the tested range, albeit marginally. The water-to-binder (W/B) ratio significantly impacts the compressive strength of the EGC. An increase in the W/B ratio from 0.28 to 0.32 reduced the compressive strength from 80.50 MPa to 71.57 MPa. In contrast, the ECC showed higher compressive strength but relatively lower strength at 3 d and 7 d. The Yingjia PVA fiber had no significant effect on the compressive strength.

#### 3.2.2. Flexural Strength

**Figure 6:**
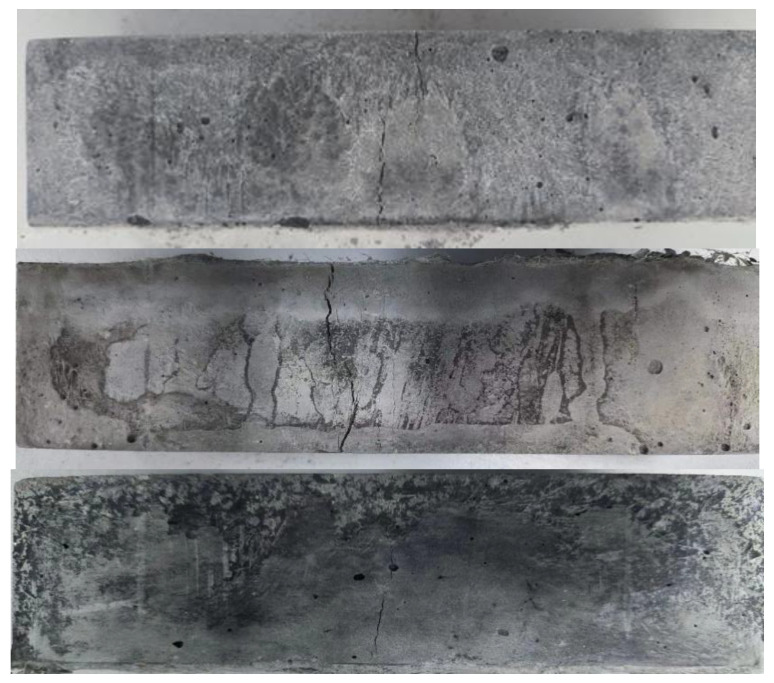
Failure mode.

Figure 6 illustrates the failure patterns of a specimen under load. The initial micro-crack emerges at the specimen’s lower end when the load reaches a certain threshold, marked by an audible cracking sound. As the load escalates, the number of cracks and their width gradually expand, accompanied by an increase in downward displacement at the mid-span of the specimen. Notably, upon failure, one crack rapidly enlarges to become the predominant crack. This behavior differs significantly from that of ordinary mortar in flexural tests; the EGC flexural specimens, despite experiencing multiple cracks, continue to sustain external loads and only cease functioning after significant deformation rather than succumbing to brittle fracture. After the damage, the specimens remain intact as a single piece, showcasing substantial energy dissipation capability despite cracks.

**Figure 7:**
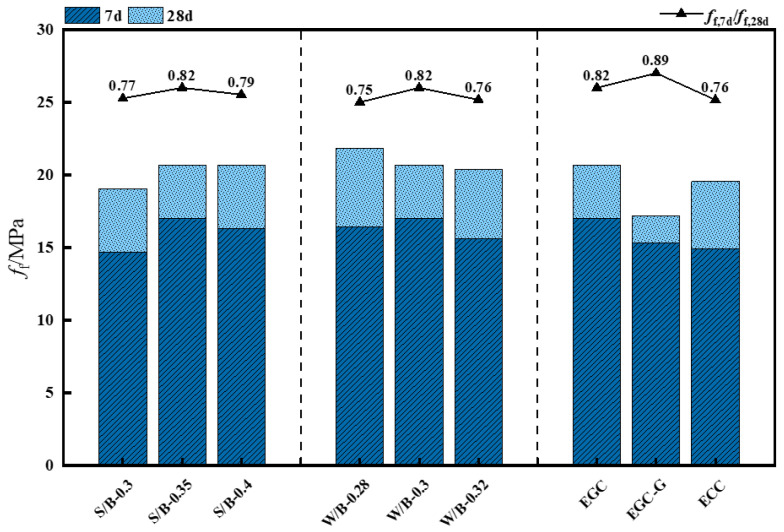
Flexural strength.

Figure 7 and Table 7 reveal that the EGC’s 7-day flexural strength exceeds 80% of its 28-day flexural strength, demonstrating high early strength. This pattern is consistent with the EGC’s compressive strength changes, indicating that the EGC has excellent early strength performance, mainly due to the geopolymers’ rapid hydration reaction and the fibers’ effective bridging action.

The trend observed in the flexural strength across varying sand-to-binder ratios mirrors the compressive strength: the flexural strength initially rises with an increase in the sand-to-binder ratio before slightly declining. This pattern is attributed to the elevated elastic modulus of quartz sand, which bolsters the flexural strength of the EGC within a specific range.

The water-to-binder ratio has a minor effect on the EGC’s flexural strength. The flexural strength decreases slightly as the ratio increases, indicating a weak negative correlation. When the ratio increases from 0.28 to 0.32, the flexural strength decreases from 21.86 MPa to 20.42 MPa, a reduction of 6.6%, less than the decrease in compressive strength.

The flexural strength significantly decreased when the fiber type was the Yingjia PVA fiber. Compared to the EGC, the flexural strength of the ECC decreased from 20.70 MPa to 19.58 MPa, with insignificant changes.

### 3.3. Uniaxial Tensile Behaviors

#### 3.3.1. Failure Patterns

During the initial loading phase, the material remains within the elastic regime, and the transition to the cracking load occurs without conspicuous signs. However, as the load increases, the specimen’s sides exhibit cracks. This is attributed to the cementitious material’s low tensile strength and pre-existing internal defects. Upon the formation of cracks, the load registered by the testing machine experiences a sudden decrease, at which point the fibers start to engage in tension, resulting in a subsequent uplift in the load curve. A clear sound of fiber rupture and pullout marks the onset of specimen cracking. With continued loading, new cracks parallel to the initial ones emerge on the specimen’s surface. Eventually, as the cracks proliferate across the entire specimen, one crack predominates by widening significantly, leading to specimen failure.

**Figure 8:**
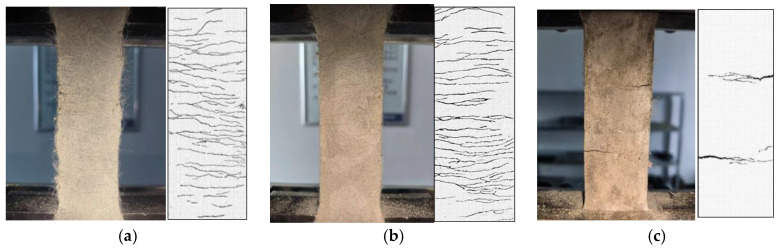
Typical failure modes: (**a**) CG, (**b**) W/B-0.32, (**c**) EGC-G.

Figure 8 gives the damage pattern and local crack development pattern of each group of specimens, and there are some differences in the damage degree of the specimens. CG and W/B-0.32 are saturated distributions of multiple fine cracks; the cracks are uniformly distributed and filled with the whole specimen, the cracks are closely adjacent, and the average width is small. The cracking area of EGC-G is denser, the integrity is poorer, and there are more uncracked areas in the area; when the peak load is reached, the dense area is more prone to expansion, and damage occurs. When the peak load is reached, the cracks in the dense area are more likely to expand and damage.

#### 3.3.2. Stress–Strain Curves

**Figure 9:**
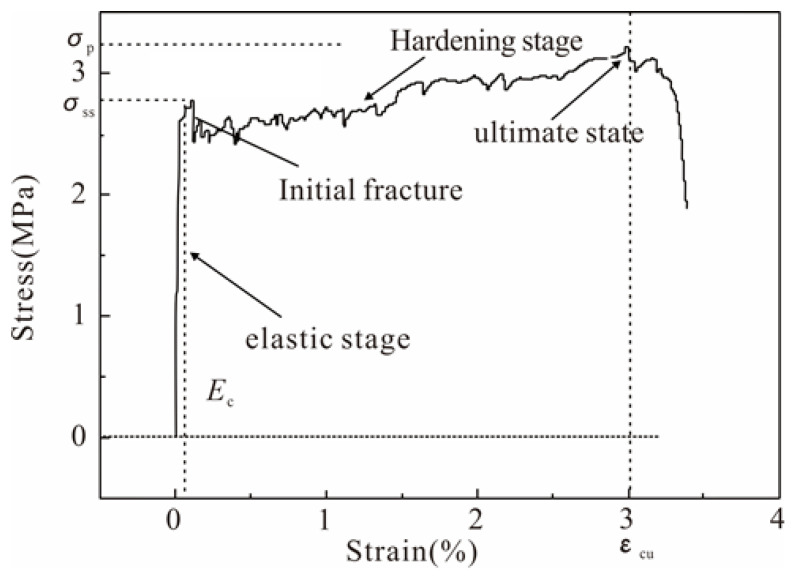
Typical stress–strain relationship.

Figure 9 illustrates the typical stress–strain curve for the Engineered Geopolymer Composite (EGC). The stress–strain behaviors of the EGC and Engineered Cementitious Composite (ECC) exhibit notable similarities. Initially, both materials undergo an elastic phase, characterized by a constant and linear slope on the curve, indicating that the base material primarily supports the stress. Following the onset of cracking, the curve exhibits a series of rises and falls, marking the transition into a hardening phase. This phase is defined by the continuous formation of new cracks and the fracture of fibers. Upon reaching crack saturation, many fibers fail, leading the specimen to its ultimate strength, at which point the curve sharply declines.

**Figure 10:**
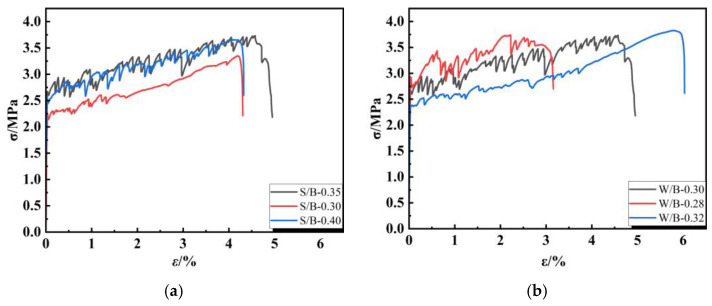

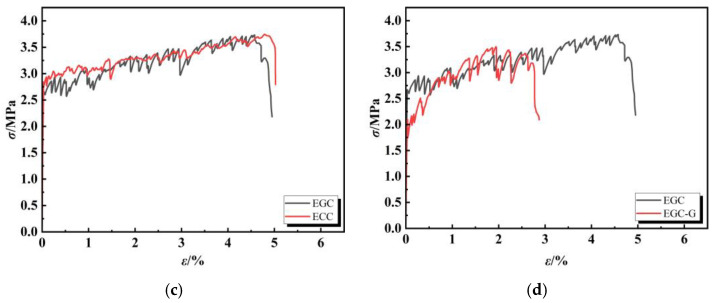
Stress–strain curve: (**a**) sand/binder ratio, (**b**) water/binder ratio, (**c**) EGC and ECC, (**d**) fiber type.

Figure 10 shows the uniaxial tensile stress–strain curves of the EGC under different variables in each group. It can be found that each specimen shows an apparent strain-hardening phenomenon. When the ratio of water to binder increases, the composite’s strain increases significantly while the strength decreases. The ratio greatly influences the flowability of the milled polymer slurry, and the enhancement of flowability contributes to the dispersion of fibers in the slurry. However, insufficient adhesion between the fibers may reduce the strain in the specimens. The peak stress in the stress–strain curve increases with the increase in the glue-to-sand ratio. The stress–strain curves of different fiber types differed significantly.

#### 3.3.3. Characteristic Parameters

Critical uniaxial tensile stress–strain curves for each specimen group were derived through a detailed analysis of the characteristic points of the uniaxial tensile performance parameters. These include the stress at first crack (*σ*_fc_), strain at first crack (*ε*_fc_), ultimate tensile stress (*σ*_tu_), ultimate tensile strain (*ε*_tu_), and the elastic modulus (*E*_c_), as documented in Table 8.

(1) Uniaxial tensile strength

**Figure 11:**
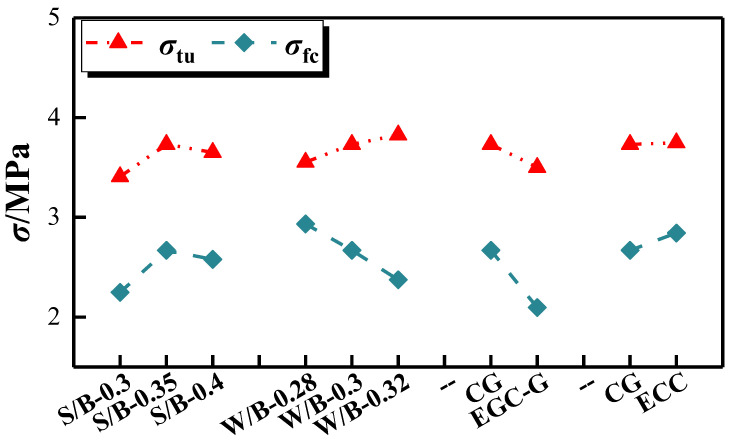
Initial cracking tensile stress and ultimate tensile stress.

Figure 11 shows the initial and ultimate tensile stress changes of the Engineered Geopolymer Composite (EGC) with different ratios. It can be seen that the initial tensile stress of the EGC decreased as the water/cement ratio increased from 0.28 to 0.30 and 0.32, with decreases of 12.4% and 23.5%, respectively. In contrast, the ultimate tensile stress increased slightly from 3.554 MPa to 3.732 MPa and 3.826 MPa. The increase in water consumption significantly impacted the strength of the reinforced section, with the stress growth rate rising from 21.1% to 61.1%. This is primarily because a lower water-to-gel ratio increases the matrix fracture toughness of the EGC, thereby increasing the stress required for the matrix to crack initially. Conversely, a higher water-to-gel ratio facilitates the dispersion of PVA fibers in the EGC matrix, increases slurry fluidity, and promotes strain-hardening characteristics, resulting in a more extended strain-hardening section and higher tensile strength at later stages. However, an excessively high water-to-cement ratio increases air bubbles in the slurry and porosity, negatively impacting strength.

The results reveal that for sand-to-binder ratios below 0.35, an increase results in both the initial cracking tensile stress and the ultimate tensile stress of the EGC. Conversely, for ratios above 0.35, the ultimate tensile stress begins to decline while the initial cracking tensile stress increases. Initially, increased sand content enhances the composite structure by providing better particle packing and a higher elastic modulus. However, when the sand-to-binder ratio becomes too high, internal porosity increases, negatively impacting the fiber bridging effect and reducing the crack site’s load-bearing capacity. Thus, incorporating a modest amount of fine sand enhances the tensile strength of the EGC, but an excessive sand content adversely affects it. This trend is attributed to higher sand-to-binder ratios, leading to increased internal porosity within the specimen, consequently diminishing the EGC’s tensile strength.

The Yingjia PVA fiber significantly reduced the non-axial tensile strength of the EGC, and the ECC’s was higher than the EGC’s.

(2) Uniaxial tensile strain

**Figure 12:**
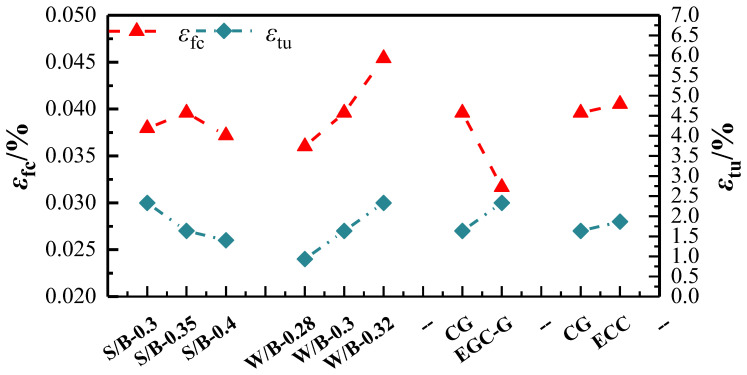
Initial cracking tensile stress and ultimate tensile stress.

Figure 12 illustrates the impact of various factors on the initial cracking tensile strain and ultimate tensile strain of the Engineered Geopolymer Composite (EGC). The ultimate tensile strains of all groups were significantly higher than the incipient tensile strains, indicating that all the EGC materials have strain-hardening behavior. Most of the ultimate tensile strains can reach more than 3%. The incipient tensile strains of the EGC materials ranged from 0.024% to 0.030%, and the ultimate tensile strains ranged from 2.724% to 5.929%.

The improvement of the deformation capacity of the EGC by increasing the hydrogel ratio is more significant. With the increase in the hydrogel ratio from 0.28 to 0.32, the initial cracking tensile strain and ultimate tensile strain increased by 25% and 58.7%, respectively. The high water-to-gel ratio can make the dispersion of PVA fibers easier, which is conducive to the multi-crack development and strain-hardening properties of the EGC.

The effect of the sand-to-binder ratios on the initial tensile strain was small. As the sand/gum ratio increased to 0.35, the ultimate tensile strain increased to a maximum of 4.574%. In contrast, the sand/gum ratio continued to increase, and the ultimate tensile strain showed a decreasing trend.

The Yingjia PVA fiber significantly reduced the ultimate tensile strain of the EGC, which has a deformability close to that of the ECC.

(3) Elastic modulus

**Figure 13:**
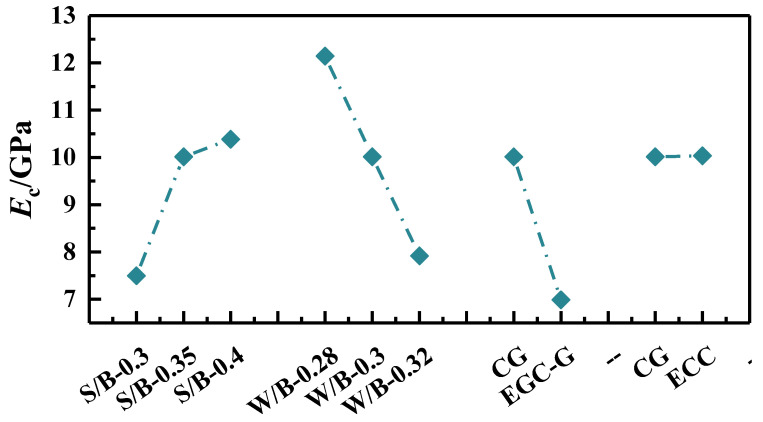
Elastic modulus.

The uniaxial tensile elastic modulus, defined as the slope of the initial linear elastic phase on the tensile stress–strain curve, offers insights into the material’s elasticity. The secant modulus, derived from the cracking stress at the curve’s linear segment’s endpoint, is the initial elastic modulus for the EGC under tension. Owing to the absence of a coarse aggregate in its composition, the tensile elastic modulus of the EGC is lower than that of conventional concrete, ranging between 6.311 GPa and 13.657 GPa. As depicted in Figure 13, an increase in the sand-to-binder ratio corresponds with a gradual rise in the elastic modulus. Conversely, an increase in the water-to-cement ratio from 0.28 to 0.32 results in a 53.3% reduction in the elastic modulus. The Yingjia PVA fiber significantly reduced the elastic modulus of the EGC, which is close to that of the ECC.

#### 3.3.4. Stress–Strain Model

Two simplified stress–strain models are commonly recognized in the analysis of fiber-reinforced cement-based composites before softening: the bilinear and trilinear models. Our tests’ stress–strain curve characteristics reveal that the curve for the Engineered Geopolymer Composite (EGC) specimens aligns with the bilinear simplified model.

This model is predicated on two fundamental assumptions.

It is posited that before reaching the initial fracture stress (*σ*_t_), the stress–strain relationship is linear, indicating elastic deformation up to point OA on the curve.

**Figure 14:**
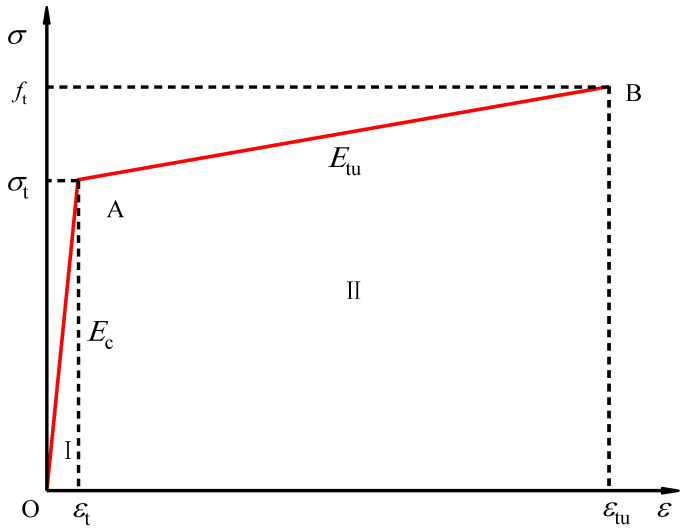
Bilinear simplified model.

Beyond the initial fracture stress, up to the ultimate stress point, the stress–strain curve enters a hardening phase, modeled as a straight line segment (AB), as illustrated in Figure 14.

The mathematical formulation of this model is presented in Equation (1):

(1) $$\sigma \left(\varepsilon \right)=\{\begin{array}{l} E_{t}\cdot \varepsilon \;\left(\varepsilon \leq \varepsilon_{t}\right)\\ \sigma_{t}+E_{\mathrm{tu}}\left(\varepsilon -\varepsilon_{t}\right)\;\left(\varepsilon_{t}<\varepsilon \leq \varepsilon_{\mathrm{tu}}\right) \end{array}$$

where *E*_t_ is the initial elastic modulus (GPa) *E*_t_ = *σ*_t_/*ε*_t_; *σ*t is the initial cracking strength (MPa); *ε*t is a preliminary cracking; *E*_tu_ is the elastic modulus (GPa) in the staged stages of the staged-hardening phase (GPa), *E*_tu_ = (*f*_tu_ − *σ*_t_)/(*ε*_t_ − *ε*_t_); *f*_tu_ is the ultimate tensile strength (MPa); and *ε*_tu_ is the ultimate tensile.

The analytical approach to simulating the strain-hardening phase connects the initial crack point to the peak stress point. However, a significant deviation has been observed between the measured stress–strain curve and this simulation, prompting the introduction of a nominal initial crack point concept. This concept utilizes the intersection points of straight lines and elastic segments, denoted by the stress (*σ*_nt_) and strain (*ε*_nt_) values, to fit the stress–strain behavior better. Consequently, by substituting the actual initial cracking point with the nominal initial cracking point, the axial tensile stress–strain relationship for the Engineered Geopolymer Composite (EGC) is accurately represented by Equation (2):

(2) $$\sigma \left(\varepsilon \right)=\{\begin{array}{l} E_{t}\cdot \varepsilon \;\left(\varepsilon \leq \varepsilon_{\mathrm{nt}}\right)\\ \sigma_{\mathrm{nt}}+E_{\mathrm{tu}}\left(\varepsilon -\varepsilon_{\mathrm{nt}}\right)\;\left(\varepsilon_{\mathrm{nt}}<\varepsilon \leq \varepsilon_{\mathrm{tu}}\right) \end{array}$$

where *σ*_nt_ is the nominal initial cracking strength, *ε*_nt_ is the nominal initial cracking, and the other symbolic meanings are the same as in Equation (1).

**Figure 15:**
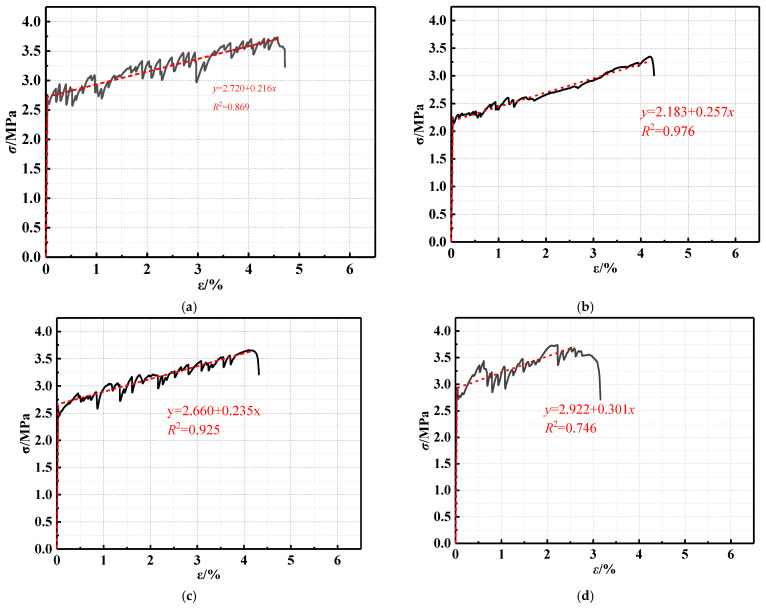

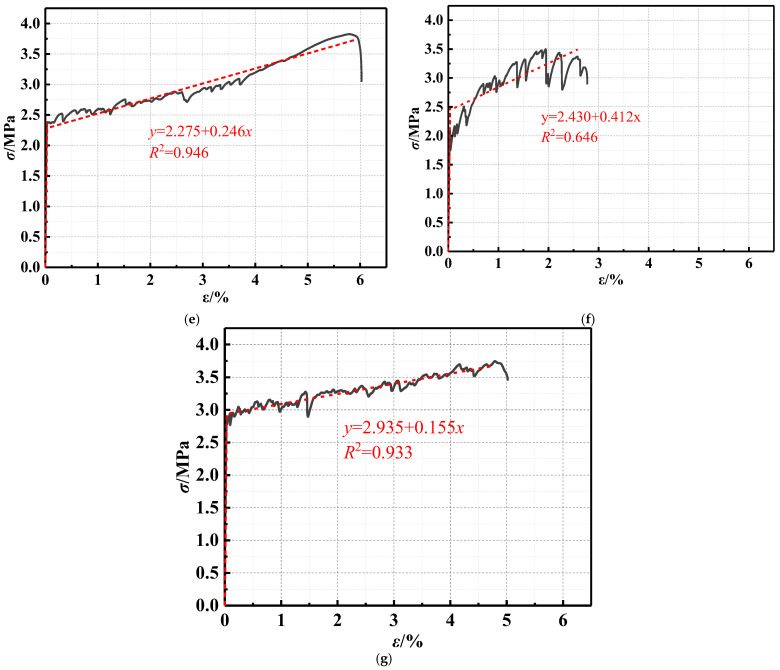
Comparison of model and test: (**a**) CG, (**b**) S/B-0.30, (**c**) S/B-0.40, (**d**) W/B-0.28, (**e**) W/B-0.32, (**f**) EGC-G, (**g**) ECC.

The Origin software 2019 was utilized for the hardening stage analysis to conduct a linear synthesis. This process involved determining the strain-hardening segment’s elastic modulus (*E*_tu_) based on the slope of the fitting line. The fitting line was then extended to intersect with the initial elastic modulus at points (*σ*_nt_, *ε*_nt_). The parameters derived from this model are presented in Table 9. A comparative analysis of the model predictions against experimental data is illustrated in Figure 15.

The analysis of the model against the experimental data, as depicted in the figure and table, demonstrates a good correlation, satisfying the requirements for computational accuracy. This outcome suggests that the employed equation is effective.

## 4. Fractal Characteristics of Cracks

### 4.1. Fractal Theory

The box-counting method calculates the fractal dimension of the specimen’s two-dimensional digital image of surface cracks. Python is used to complete the crack image’s grayscale, binarization, and noise reduction processing. The box size range is set according to the image resolution, and the optimal range is selected based on the linear fitting effect. Repeat covering and counting *N* (*r*) on crack maps is performed using cubes with different side lengths r. According to fractal theory, the relationship between *N* (*r*) and *r*, as well as the fractal dimension *D*_c_, is as follows:

(3) $$N\left(r\right)\propto r^{-D_{c}}$$

After taking the commonly used logarithms from both sides of the above equation, it can be concluded that

(4) $$\mathrm{lg}N\left(r\right)\propto -D_{c}\mathrm{lg}r$$

**Figure 16:**
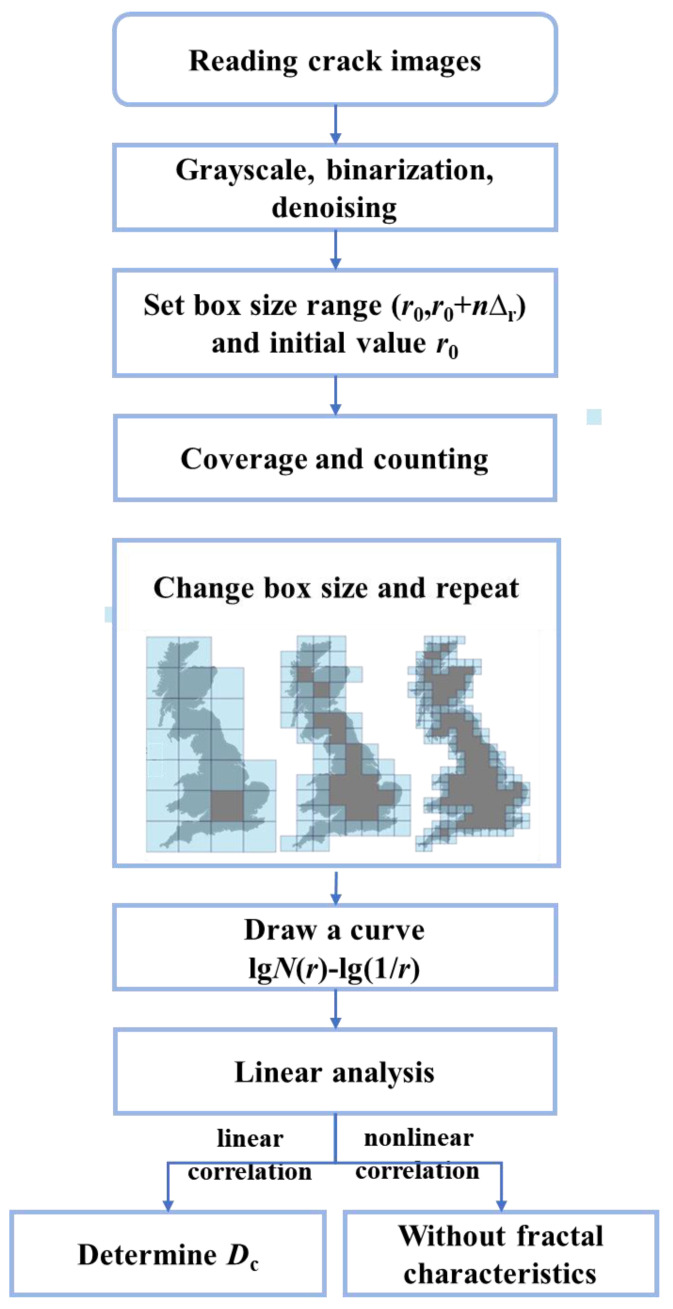
The principle and steps of the box-counting method.

The slope of fitting the logarithm lg*N* (*r*) and lg (1/*r*) is the fractal dimension *D*_c_ of the crack surface. The principle and calculation steps of the box-counting method are shown in Figure 16.

### 4.2. Experimental Data Collection

**Figure 17:**
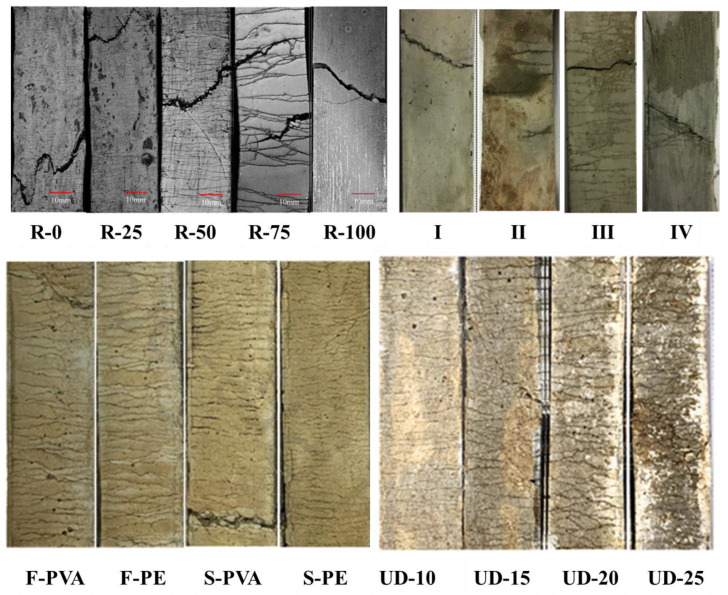
Fractal dimension of a crack.

A database of measurements was compiled from published past studies [25,26,27,28,29]. The database includes 17 EGC samples. The basic design variables, such as precursor, alkali activator, recycled aggregate, and fiber of GRC, were selected as input variables by considering the failure mechanism, as listed in Table 10 and Figure 17.

**Figure 18:**
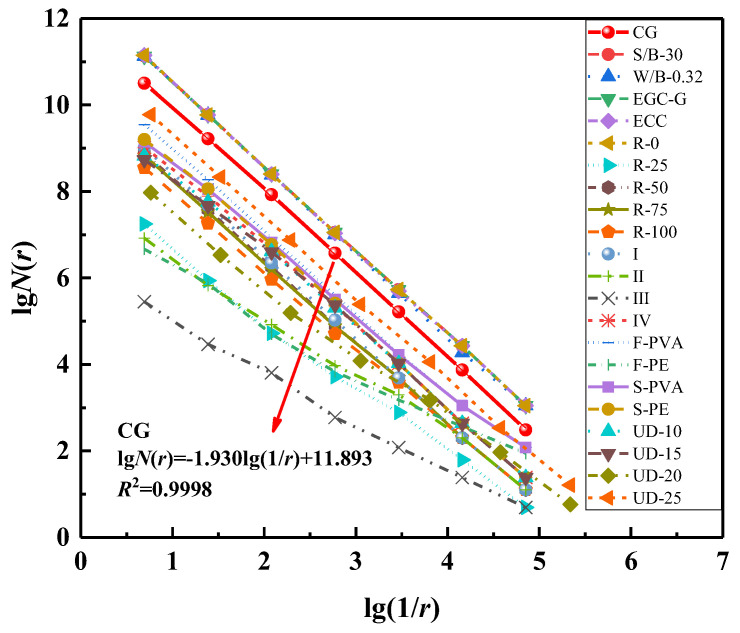
The figure of lg*N*(*r*) and lg(1/*r*).

Figure 18 shows a strong correlation between each tensile specimen’s lg*N* (*r*) and lg (1/*r*), indicating that their crack fractal characteristics are apparent. The fractal dimension *D*_c_ and correlation coefficient R^2^ of each specimen are given. The fractal dimension of each specimen is 1.523~1.973, which characterizes the crack surface’s roughness and reflects the crack’s propagation behavior. The high fractal dimension of the specimen indicates that the more irregular the crack surface, the higher the complexity of the crack path, and the material’s toughness, energy absorption characteristics, and crack resistance can be fully utilized.

### 4.3. Fractal Dimension

**Figure 19:**
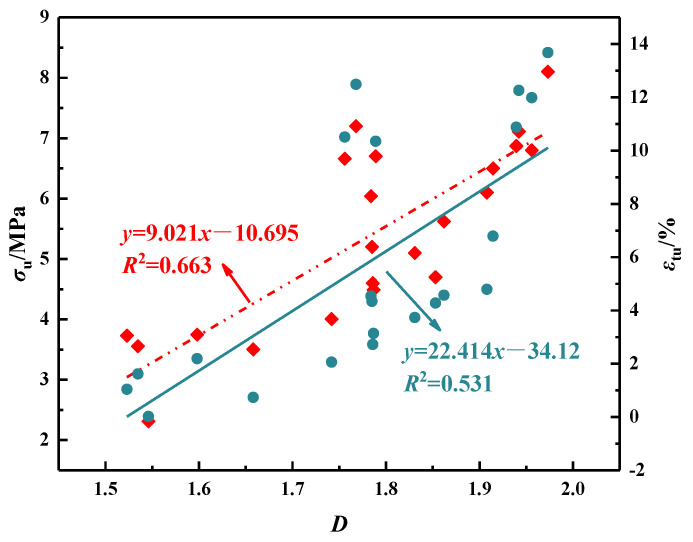
The relationship between the flexural performance parameters and fractal dimension.

Figure 19 shows an excellent linear relationship between the fractal dimension and the ultimate stress and strain of the specimen, with correlation coefficients of 0.663 and 0.531, respectively, indicating that the fractal dimension well describes the relationship between crack morphology and the axial tensile performance index of the specimen. According to fractal theory, the larger the fractal dimension, the more complex the distribution and propagation path of cracks. A complex crack network can absorb and dissipate more energy, improving the flexibility of the specimen.

## 5. Conclusions

The effects of the GGBS replacement rate, water/binder ratio, and fiber volume content on the working and mechanical properties of the Engineered Geopolymer Composite (EGC) were investigated. The following conclusions can be drawn:

(1) The sand-to-binder ratio significantly influences the rheological properties of the EGC; an increase in this ratio enhances rheology, whereas a decrease tends to diminish it. Additionally, a lower water-to-binder ratio was found to substantially reduce rheology, indicating that fluidity and workability are critically dependent on the precise balance of these ratios, affecting the ease of application and quality of the final product.
(2) The compressive strength of the EGC specimens at seven days reached 80% to 92% of their 28-day strength, indicating a rapid early strength gain. The hierarchy of factors influencing compressive strength was identified as the fiber type having the greatest impact, followed by the water-to-binder ratio and then the sand-to-binder ratio.
(3) The stress–strain curves for both the EGC and Engineered Cementitious Composite (ECC) displayed similar characteristics, predominantly in the pre-cracking elastic stage where the matrix material sustains most of the applied stress, maintaining a consistent and predictable deformation pattern. This similarity underscores the potential of the EGC as a viable alternative to the ECC with comparable mechanical resilience.
(4) An increase in the water/binder ratio leads to higher ultimate strain capacities in the composite materials, albeit at the expense of reduced strength, demonstrating a trade-off between ductility and strength. This study has established a quantifiable stress–strain relationship for EGC, tailored to different mix ratios.
(5) Future research should address the long-term durability and economic viability of PVA-EGCs, exploring their performance across a broader range of environmental conditions and material compositions to enhance their practical application in the construction industry.

## Figures and Tables

**Table 1:** Properties of the raw materials.

Material	SiO_2_	Al_2_O_3_	Fe_2_O_3_	CaO	K_2_O	MgO	Na_2_O	D50 Size/μm
Fly ash	54.06	28.26	4.52	6.27	1.84	1.29	0.91	23.2
GGBS	32.08	15.13	0.47	38.61	0.43	8.45	0.49	24.0

**Table 2:** Basic properties of PVA fibers.

Material	Length/mm	Diameter/μm	Ultimate Strength/MPa	Elasticity Modulus/GPa	Elongation/%	Oil Coverage Rate/%
REC15-type PVA	12	39	1620	42.8	6.0	0.8
Yingjia PVA	12	38	1600	40.0	6.5	-

**Table 3:** Basic properties of quartz sand.

Material	Particle Size/μm	Packing Density/(g∙cm^−3^)	Appearance Density/(g∙cm^−3^)	Breakage Rate/%	Wear Rate/%	Porosity/%	Hardness/%
Quartz sand	100–200	2.66	1.75	0.51	0.35	43	7.5

**Table 4:** Mix proportions of EGC.

Specimens	FA /(kg∙m^−3^)	GGBS /(kg∙m^−3^)	Concrete /(kg∙m^−3^)	Quartz Sand /(kg∙m^−3^)	Water /(kg∙m^−3^)	Alkali Activator /(kg∙m^−3^)	Fiber Volume Content /(kg∙m^−3^)	Fiber Type
CG	923.2	230.8	-	403.9	86.5	432.7	26.0	K
S/B-0.3	943.6	235.9	-	353.9	88.5	442.3	26.0	K
S/B-0.4	903.6	225.9	-	451.8	84.7	423.5	26.0	K
W/B-0.28	945.0	236.2	-	413.4	65.0	443.0	26.0	K
W/B-0.32	902.3	225.6	-	394.8	107.2	423.0	26.0	K
EGC-G	923.2	230.8	-	403.9	86.5	432.7	26.0	G
ECC	-	-	1236.2	432.7	370.9	-	26.0	K

Note: S/B-0.3 indicates that the sand/cement ratio is 0.3; W/B-0.28 indicates that the water/cement ratio is 0.28; EGC-G indicates that the type of fiber is domestic fiber; ECC indicates that the cementitious material is cement.

**Table 5:** Rheological property (cm).

Specimens	D_1_	D_2_	Average Value	SE
CG	16.7	16.9	16.80	0.07
S/B-0.3	17.2	17.5	17.35	0.11
S/B-0.4	15.3	15.4	15.35	0.04
W/B-0.28	14.9	14.7	14.80	0.07
W/B-0.32	16.9	17.3	17.10	0.14
EGC-G	16.5	16.2	16.35	0.11
ECC	17.2	17.3	17.25	0.04

Note: SE is Standard Error.

**Table 6:** Compressive strength (MPa).

Specimens	*f*_cu,3d_	SE	*f*_cu,7d_	SE	*f*_cu,28d_	SE
CG	72.37	0.85	74.98	1.71	76.53	1.64
S/B-0.3	68.12	1.61	70.59	1.61	74.92	1.77
S/B-0.4	73.29	1.73	76.08	1.73	79.08	1.81
W/B-0.28	80.95	1.91	82.86	1.91	86.50	1.84
W/B-0.32	66.71	1.57	67.80	1.57	71.57	1.69
EGC-G	67.98	1.60	72.81	1.60	80.91	1.91
ECC	31.82	0.75	45.29	0.75	88.14	2.08

**Table 7:** Flexural strength.

Mixtures	*f*_f,7d_/MPa	SE	*f*_f,28d_/MPa	SE
CG	17.00	0.40	20.70	0.49
S/B-0.3	14.70	0.35	19.08	0.45
S/B-0.4	16.32	0.38	20.70	0.49
W/B-0.28	16.44	0.39	21.86	0.52
W/B-0.32	15.61	0.37	20.42	0.48
EGC-G	15.34	0.36	17.20	0.41
ECC	14.94	0.35	19.58	0.46

Note: *f*_f,7d_ and *f*_f,28d_ are the flexural strengths of the EGC at 7 d and 28 d, respectively.

**Table 8:** Uniaxial tensile property test results.

Mixtures	*σ*_fc_/MPa	*ε*_fc_/%	*σ*_tu_/MPa	*ε*_tu_/%	*E*_c_/GPa
CG	2.670	0.027	3.732	4.574	10.013
S/B-0.3	2.249	0.030	3.409	4.190	7.497
S/B-0.4	2.579	0.025	3.652	4.008	10.385
W/B-0.28	2.934	0.024	3.554	3.736	12.141
W/B-0.32	2.375	0.030	3.826	5.929	7.917
EGC-G	2.096	0.030	3.501	2.724	6.987
ECC	2.843	0.028	3.747	4.792	10.034

**Table 9:** Tensile constitutive model parameters.

Mixtures	*σ*_nt_/MPa	*ε*_nt_/%	*E*_c_/GPa	*E*_tu_/GPa	*R* ^2^
CG	2.726	0.0272	10.013	0.216	0.869
S/B-0.3	2.191	0.0292	7.497	0.257	0.976
S/B-0.4	2.666	0.0257	10.385	0.235	0.925
W/B-0.28	2.929	0.0241	12.141	0.301	0.746
W/B-0.32	2.282	0.0288	7.917	0.246	0.946
EGC-G	2.444	0.0350	6.987	0.412	0.646
ECC	2.939	0.0293	10.034	0.155	0.933

**Table 10:** Detailed information of the database.

	Specimen	*σ*_u_/MPa	*ε*_tu_/%
Peng	R-0	6.66	4.28
	R25	6.63	4.34
	R-50	5.62	4.54
	R-75	4.49	2.19
	R-100	2.31	0.0196
Ling	I	4.7	3.14
	II	5.1	1.62
	III	6.8	1.04
	IV	6.1	0.74
Nguyễn	F-PVA	6.64	10.51
	F-PE	6.04	6.79
	S-PVA	5.18	11.99
	S-PE	4.60	10.35
Nguyễn	UD-10	7.11	12.49
	UD-15	6.79	13.68
	UD-20	8.10	12.26
	UD-25	7.18	10.88

## Data Availability

The data presented in this study are available on request from the corresponding author.
